# Heterogeneous Effects of Fibroblast-Myocyte Coupling in Different Regions of the Human Atria Under Conditions of Atrial Fibrillation

**DOI:** 10.3389/fphys.2019.00847

**Published:** 2019-07-04

**Authors:** Jorge Sánchez, Juan F. Gomez, Laura Martinez-Mateu, Lucia Romero, Javier Saiz, Beatriz Trenor

**Affiliations:** Centre for Research and Innovation in Bioengineering, Universitat Politècnica de València, Valencia, Spain

**Keywords:** atrial fibrillation, computer simulation, structural remodeling, myofibroblast, vulnerability

## Abstract

**Background:** Atrial fibrillation (AF), the most common cardiac arrhythmia, is characterized by alteration of the action potential (AP) propagation. Under persistent AF, myocytes undergo electrophysiological and structural remodeling, which involves fibroblast proliferation and differentiation, modifying the substrate for AP propagation. The aim of this study was to analyze the effects on the AP of fibroblast-myocyte coupling during AF and its propagation in different regions of the atria.

**Methods:** Isolated myocytes were coupled to different numbers of fibroblasts using the established AP models and tissue simulations were performed by randomly distributing fibroblasts. Fibroblast formulations were updated to match recent experimental data. Major ion current conductances of the myocyte model were modified to simulate AP heterogeneity in four different atrial regions (right atrium posterior wall, crista terminalis, left atrium posterior wall, and pulmonary vein) according to experimental and computational studies.

**Results:** The results of the coupled myocyte-fibroblast simulations suggest that a more depolarized membrane potential and higher fibroblast membrane capacitance have a greater impact on AP duration and myocyte maximum depolarization velocity. The number of coupled fibroblasts and the stimulation frequency are determining factors in altering myocyte AP. Strand simulations show that conduction velocity tends to homogenize in all regions, while the left atrium is more likely to be affected by fibroblast and AP propagation block is more likely to occur. The pulmonary vein is the most affected region, even at low fibroblast densities. In 2D sheets with randomly placed fibroblasts, wavebreaks are observed in the low density (10%) central fibrotic zone and when fibroblast density increases (40%) propagation in the fibrotic region is practically blocked. At densities of 10 and 20% the width of the vulnerable window increases with respect to control but is decreased at 40%.

**Conclusion:** Myocyte-fibroblast coupling characteristics heterogeneously affect AP propagation and features in the different atrial zones, and myocytes from the left atria are more sensitive to fibroblast coupling.

## Introduction

Atrial fibrillation (AF) is the most common cardiac arrhythmia and is becoming more prevalent with population aging (Iwasaki et al., 2011). In persistent atrial fibrillation (PeAF), lasting longer than 7 days (Kirchhof et al., 2016), spontaneous, pharmacological, or ablative resumption of sinus rhythm is infrequent with prompt recurrences or commonly failed cardioversions (Jalife and Kaur, 2015).

In PeAF, cardiac tissue experiences structural and electrical remodeling driven by changes of the extracellular matrix, fibroblast differentiation, fatty and inflammatory infiltration, and ion channel remodeling, among other alterations (Kirchhof et al., 2016). The heterogeneities in the atrial substrate cause fiber discontinuities and local conduction disturbances favoring re-entries (Nattel and Dobrev, 2017).

The atria have a complex anatomical structure, differences between the right and left atrium, and complex structures like the pulmonary vein or left and right appendages (Sanchez-Quintana et al., 2012; Ferrer et al., 2015). They all have their own electrophysiological characteristics, which determine specific action potential (AP) waveforms and conduction velocities (CV), which are crucial to AF pathophysiology.

Approximately 75% of the adult myocardium tissue volume is occupied by myocytes, accounting for only 30–40% of the total number of cells (Biernacka and Frangogiannis, 2011). Fibroblasts are considered as the predominant non-myocyte cell. They become active under inflammatory response and then differentiate into myofibroblasts (Chacar et al., 2017) by different pathways (e.g., oxidative stress, atrial dilatation, calcium overload, and inflammation), although the precise mechanisms have not yet been established (Jalife and Kaur, 2015). Myofibroblasts differ from fibroblasts in that they develop contractile proteins and exhibit a more depolarized resting membrane potential (Salvarani et al., 2017) and greater membrane capacitance (Sridhar et al., 2017). The differences in resting membrane potential and membrane capacitance between fibroblasts and myofibroblasts might have an important repercussion on the electrophysiology of coupled myocytes.

Recent experimental studies have shown that fibroblasts express voltage-dependent sodium channels allowing an inward current (I_Na_) (Chatelier et al., 2012; Koivumäki et al., 2014a; Poulet et al., 2016). Although they are not electrically excitable they may affect the myocytes’ electrophysiological properties (Gaudesius et al., 2003; Kohl et al., 2005; Miragoli et al., 2006). Several animal experimental models have shown that fibroblasts electrically couple to myocytes and alter their AP (Rook et al., 1992; Gaudesius et al., 2003; Burstein et al., 2007; Rohr, 2011; Jalife and Kaur, 2015; Jousset et al., 2016; Quinn et al., 2016). Kohl et al. (1994) found that fibroblasts reacted electrically with myocytes through gap junctions and this was later corroborated *in vitro* in several animal species (Gaudesius et al., 2003; Camelliti et al., 2004; Kohl et al., 2005). However, the extent of (*in vivo*) fibroblast-myocyte electrotonic coupling in native myocardium remains controversial (Kohl and Gourdie, 2014). Recently, a study by Quinn et al. (2016) showed the existence of tunneling nanotube connections between excitable and non-excitable cells (e.g., myofibroblasts) in cardiac scar border tissue at the border zone of the scar in mice hearts. Similarly, Burstein et al. (2007) observed fibroblasts proliferation and differentiation into myofibroblasts during AF *in vivo* canine hearts. Fibroblast proliferation has also been observed in human hearts (Krul et al., 2015; Fukumoto et al., 2016), in regions with altered conduction velocity, although electrical coupling has not been demonstrated.

Computational simulations of the cellular electrophysiology have become a powerful tool for studying the role that fibroblasts play in the atrial substrate (MacCannell et al., 2007; Maleckar et al., 2009; Koivumäki et al., 2014a). Their flexibility permits modifying the cellular electrophysiology and varying different characteristics to analyze the impact of electrotonic coupling with electrically remodeled myocytes in PeAF (McDowell et al., 2013; Morgan et al., 2014; Sridhar et al., 2017). MacCannell et al. (2007) formulated an active and a passive model for fibroblasts electrophysiology, considering a capacitance and an ohmic resistance in the first case and a capacitance and four ionic currents in the second case. The active model is more realistic and has a major impact on myocytes, this is why in the present study the active model is implemented. Simulations also offer the possibility of studying how fibroblasts can change arrhythmia dynamics by reducing CV and introducing heterogeneities in the atrial substrate (Tanaka et al., 2007; Saha et al., 2018).

The present simulation study analyzes the effects of fibroblast-myocyte coupling in PeAF in specific structurally and electrophysiologically remodeled atrial tissues and compares the effect of fibroblasts and myofibroblasts on myocyte AP features, tissue conduction velocity and vulnerability to re-entries.

## Materials and Methods

### Myocyte Electrophysiological Models

The human atrial AP model proposed by Koivumäki et al. (2011) was used to simulate atrial APs. To reproduce the tissue electrophysiology of different anatomical atrial regions, we modified the conductance of five ionic currents: transient outward K^+^ current (I_to_), potassium rapid current (I_Kr_), potassium slow current (I_Ks_), time independent K^+^ current (I_K1_), and L-type Ca^2+^ current (I_CaL_) as proposed in previous simulation studies (Krueger et al., 2012; Ferrer et al., 2015). The conductance values, shown in Table 1, were modified according to the experimental values to reproduce the AP waveform in all four regions: right atrium posterior wall (RA), crista terminalis (CT), left atrium posterior wall (LA), and the pulmonary vein (PV) areas.

**Table 1:** Ionic conductance modifications.

Channel conductance	RA	LA	PV	CT
*g_to_*	1.0	1.0	0.25	0.55
*g_Kr_*	1.0	1.6	4.8	0.55
*g_CaL_*	1.0	0.67	0.3	1.0
*g_K1_*	1.0	1.0	0.7425	1.0
*g_Ks_*	1.0	1.0	5.12	1.0

*Relative values for each atrial region (right atrium -RA-, left atrium -LA-, pulmonary veins -PV- and crista terminalis -CT-) with respect to the maximum conductance of the potassium transient outward current (g_*to*_), potassium rapid current (g_*Kr*_), calcium L-type current (g_*CaL*_), and potassium slow current (g_*Ks*_) in the Koivumäki et al. (2011) model*.

### Ionic Remodeling

Table 2 shows the values used to introduce AF electrical remodeling in all atrial regions by modifying ion channel conductances for I_CaL_, I_to_, I_K1_, sustained outward K^+^ current (I_sus_), Na^+^/Ca^2+^ exchanger (NCX), sarcoplasmic reticulum Ca^2+^ ATPase (SERCA) pump, and ryanodine receptors (RyR), and specific calcium handling parameters, such as phospholamban (PLB), sarcolipin (SLN), and the baseline phosphorylation (phos). Dilation was also modeled by increasing the length of the cell by a factor of 1.1 (see Table 2) as described in Koivumäki et al. (2014b).

**Table 2:** Atrial fibrillation electrical remodeling.

L_cell_	g_CaL_	g_to_	g_sus_	g*_K_*_1_	*K*_NaCa_	cpumps	PLB	SLN	phos	RyR
1.1	0.41	0.38	0.62	1.62	1.5	0.84	1.18	0.6	2	2

*Based on experimental observations/prior computational works (Koivumäki et al., 2014b), to account for persistent atrial fibrillation remodeling the ionic and cellular parameters modified were: cell length (L_*cell*_), conductance of calcium L-type current (g_*CaL*_), conductance of potassium transient outward current (g_*to*_), conductance of potassium sustained current (g_*sus*_), conductance of potassium inward rectifier (g_*K1*_), maximum conductance of Na+/Ca2+ exchanger (K_*NaCa*_), conductance of sarcoplasmic reticulum Ca2+ ATPase (SERCA) pump (cpumps), phospholamban (PLB), sarcolipin (SLN), baseline phosphorylation (phos), and ryanodine receptors (RyR). The values indicate the factor by which the original values are multiplied*.

### Fibroblast Electrophysiological Model

Fibroblast electrophysiology was represented according to the Koivumäki et al. (2014a) model. Membrane sodium current was updated to match recent experimental results (Poulet et al., 2016) (see Supplementary Table S1). The uncoupled fibroblast resting membrane potential (RMP_f_) was set to -45 or -26 mV, as suggested by experimental data (Poulet et al., 2016; Salvarani et al., 2017). These values were obtained by shifting the gating variable for the time dependent potassium current as in previous simulation studies (Maleckar et al., 2009; Morgan et al., 2016), and shown in Supplementary Table S2. The fibroblast membrane capacitance (C_mf_) values were varied within experimental ranges (6.3 and 50.4 pF) (MacCannell et al., 2007; Xie et al., 2009; McArthur et al., 2015; Poulet et al., 2016; Sridhar et al., 2017). Following previous experimental and simulation studies, we set RMP_f_ to -45 mV and C_mf_ to 6.3 pF for atrial fibroblasts (MacCannell et al., 2007; Maleckar et al., 2009), and a more depolarized RMP_f_ of -26 mV and a C_mf_ of 50.4 pF for atrial myofibroblasts (Morgan et al., 2016; Poulet et al., 2016; Salvarani et al., 2017; Sridhar et al., 2017). The code is available and can be found at http://hdl.handle.net/10251/120395.

### Cellular Simulations (0D)

To compute the membrane potential in a cell, Equations (1, 2, and 3) were used:

$${\text{C}}_{\text{myo}}\frac{{\text{dV}}_{\text{myo}}}{\text{dt}}\;\text{+}\;{\text{I}}_{\text{ion-myo}}\;\text{+}\;{\text{I}}_{\text{gap}}\;\text{=}\;\text{0}\;\;\;\;\;\;\;\left(\text{1}\right)$$

$${\text{C}}_{\text{fib}}\frac{{\text{dV}}_{\text{fib}}}{\text{dt}}\;\text{+}\;{\text{I}}_{\text{ion-fib}}\;\text{-}\;{\text{I}}_{\text{gap}}\;\text{=}\;\text{0}\;\;\;\;\;\;\;\left(\text{2}\right)$$

$${\text{I}}_{\text{gap}}\;\text{=}\;\sum_{\text{i}=0}^{\text{n}}{\text{G}}_{\text{gap}}\;\left({\text{V}}_{\text{myo}}\;\text{-}\;{\text{V}}_{{\text{fib}}_{\text{i}}}\right)\;\;\;\;\;\;\left(\text{3}\right)$$

where C_myo_ is the membrane capacitance of the myocyte, I_ion-myo_ is the total ionic current flowing through ionic channels of the myocyte, V_myo_ is the membrane potential in the myocyte, C_fib_ is the membrane capacitance of the fibroblast, I_ion-fib_ is the total ionic current flowing through ionic channels of the fibroblast, V_fib_ the membrane potential in the fibroblast, I_gap_ is the total ionic current flowing through the gap junction, n is the number of coupled fibroblasts to one myocyte and G_gap_ is the coupling conductance between myocyte and fibroblast. G_gap_ was set to 0.5 nS (Morgan et al., 2016), a value within the range of 0.3–8 nS measured in cultured myocyte-fibroblast experiments as used in previous simulation studies (MacCannell et al., 2007; Rook et al., 1992). The number of fibroblasts coupled to a single myocyte varied from 0 to 9 (Koivumäki et al., 2014a), as shown in Supplementary Figure S1.

Stabilization of the cellular model of each region was achieved after 1000 stimuli of 2 ms duration and an amplitude of twice the threshold value. After this number of stimuli, APD_90_ values and ion concentration ranges reached steady state. The basic cycle length (BCL) was 1 s, 250 ms, or 2 s.

The following features of the myocyte AP were measured: AP duration at 90% repolarization (APD_90_), depolarization upstroke velocity measured as the maximum variation of the depolarization phase (dV/dt_max_), and the RMP.

### Strand and Tissue Simulations

The diffusion-reaction Eq. (4) of the monodomain formalism was used to simulate AP electrical propagation:

$$\nabla \;\cdot \;\left(D\nabla \text{V}\right)\;=\;C_{m}\frac{\text{dV}}{\text{dt}}\;+\;I_{ion}\;\;\;\;\;\;\left(4\right)$$

where D is the diffusion tensor, C_m_ is the membrane capacitance, and I_ion_ stands for ionic currents through the membrane. Diffusion coefficients were adjusted for the different atrial regions to achieve physiological CVs along a strand of myocytes in this particular tissue.

Diffusion coefficients for myocytes (Dm) were calculated and adjusted to achieve physiological CVs, yielding a Dm of 3.84 cm^2^/s in RA and LA (CV = 70 cm/s), a Dm of 6.384 cm^2^/s in CT (CV = 100 cm/s), and a Dm of 4.081 cm^2^/s in PV (CV = 80 cm/s). In PeAF, the diffusion coefficient was reduced by 50% to reproduce gap junction remodeling (Harrild and Hen, 2000; Ten Tusscher et al., 2004; Krogh-madsen et al., 2012; McDowell et al., 2012).

For one-dimensional simulations (1D), a strand with a space discretization of 100 μm was used for myocytes (Satoh et al., 1996; Courtemanche et al., 1998; Nygren et al., 1998; Sachse et al., 2008) and 10 μm for fibroblasts-myofibroblasts (Sachse et al., 2008; Xie et al., 2009; Brown et al., 2015). Fibroblasts-myofibroblasts were randomly distributed along the strand at different densities (10, 20, and 40%) using a uniform random probabilistic function. One hundred random distributions were generated for each density value (see Supplementary Figure S3). The strand was paced with a BCL of 1 s for 50 s. The first element of the strand was stimulated with an amplitude of twice its threshold. CV was measured in the 50th pulse. The diffusion coefficient for elements with fibroblasts was halved with respect to the myocyte elements (Vasquez et al., 2004; Gomez et al., 2014b; Morgan et al., 2016).

Two-dimensional (2D) meshes representing cardiac tissues for RA and LA were built with a central fibrotic region of 2 cm diameter with different randomly distributed myofibroblast densities. Ten random distributions were implemented for each density (see Supplementary Figures S1, S3). The tissue grid had 524 × 524 elements with a spatial resolution of 100 μm. An anisotropic ratio of 2.86:1 was considered in RA and LA (Ferrer et al., 2015).

To study the effect of myofibroblasts on vulnerability to reentry, reentrant activity was generated using cross-field stimulation protocol S1-S2. S1 stimuli consisted of 10 pulses applied to the left border of the computational mesh to stabilize the tissue and S2 was applied to the bottom left corner of the tissue.

In order to obtain the instantaneous location of the rotors, we used phase singularity (PS) detections (Sabir et al., 1974; Gray et al., 1998; Gomez et al., 2014a; Martinez-Mateu et al., 2018) first building phase maps based on the Hilbert transform (HT) of the APs (Warren et al., 2003; Yamazaki et al., 2012) (5) by computing the instantaneous phase θ (6), whose values ranged from -π to π radians:

$$\text{HT}\left[V\left(\text{t}\right)\right]\;=\;\frac{1}{\text{ π}}\;\cdot \;\int_{-\infty }^{\infty }\;\frac{\text{V}\left(\text{τ}\right)}{\text{t}\;-\;\text{τ}}\;\cdot \;\text{dτ}\;\;\;\;\;\;\;\left(5\right)$$

$$\text{θ}\;=\;{\text{tan}}^{-1}\;\left(\frac{\text{HT}\left[\text{V}\left(\text{t}\right)\right]}{\text{V}\left(\text{t}\right)}\right)\;\;\;\;\;\;\;\;\;\left(6\right)$$

where V is the membrane potential. Then PSs, where all phases converge (7), were computed to track the rotor trajectory:

∮∇θdr = ±2π   (7)

Phase singularity detections were also used to assess heterogeneity degree in the tissue due to the inclusion of fibrotic regions.

### Implementation

The differential equations of the cellular models were solved using the Rush-Larsen method for the gating variables and the forward Euler method for all other ordinary differential equations with a time step of 10 μs. The monodomain Eq. (4) was solved using the finite elements method with “no flux” boundary condition.

Simulations were implemented in C++ and CUDA languages. Computations were performed on an Intel(R) Xeon(R) CPU E5-2603v3 processor with an NVIDIA Tesla K40c graphic card (see Supplementary Figure S2).

## Results

### Cellular Simulations

**Figure 1:**
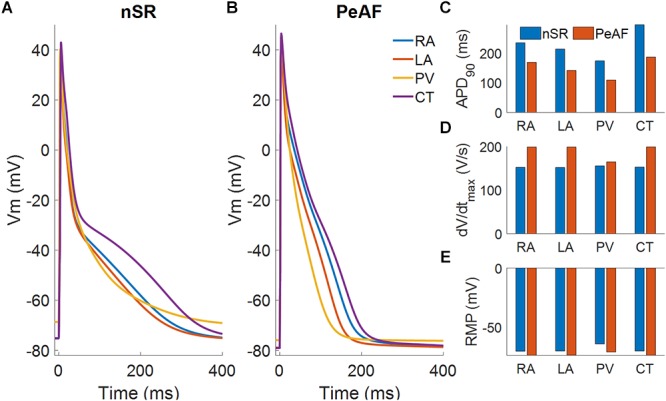
Myocyte action potentials from four different regions of the atria: right atria (RA), left atria (LA), pulmonary vein (PV), and crista terminalis (CT). **(A)** Myocyte action potentials under normal sinus rhythm (nSR). **(B)** Myocyte action potentials under the effect of electrical remodeling during persistent atrial fibrillation (PeAF). **(C)** Myocyte action potential duration (APD_90_) under nSR and PeAF. **(D)** Myocyte action potential maximum upstroke velocity (dV/dt_max_) under nSR and PeAF. **(E)** Myocyte action potential resting membrane potential (RMP) under nSR and PeAF.

Figure 1 shows the APs obtained in cellular simulations with a BCL of 1000 ms under normal sinus rhythm (Panel A) and PeAF (Panel B) conditions, highlighting the differences in APD_90_ for each atrial region. Under physiological conditions, the APD_90_ was 236.4 ms for RA, 214 ms for LA, 176.1 ms for PV, and 292.8 ms for CT. However, under PeAF conditions APD_90_ was 165.5 ms, 139.8 ms, 120.8 ms, and 180.2 ms for the RA, LA, PV, and CT, respectively. APD_90_ decreased by 40% for RA, LA, and CT and by approximately 30% for PV with respect to normal conditions (see Panel C). In PaAF, in addition to APD shortening, RMP dropped from -75 to -79 mV, and dV/dt_max_ increased from 163 V/s to 168 V/s for RA, CT, and LA. RMP fell from -68 to -78 mV, and dV/dt_max_ rose from 157 V/s to 165 V/s for PV (see Panels E and D).

**Figure 2:**
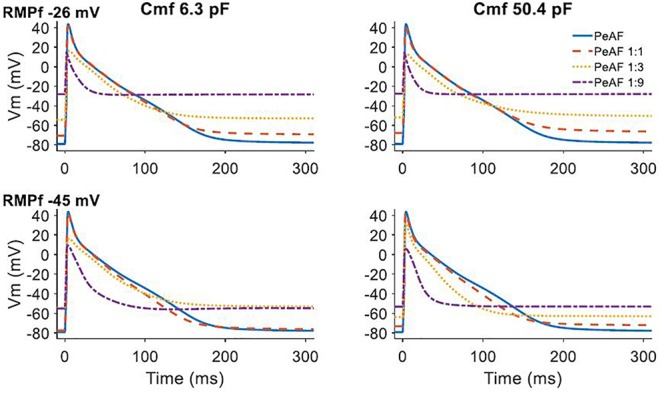
Effect of fibroblast and myocyte coupling under persistent atrial fibrillation (PeAF) electrical remodeling in RA (baseline model). First and second rows show fibroblasts resting membrane potential (RMPf) of –26 and –45 mV, respectively, for a fibroblast membrane capacitance (Cmf) of 6.3 pF (first column) and 50.4 pF (second column). The different traces are action potentials of isolated myocytes under PeAF (blue), one myocyte under PeAF coupled to 1 fibroblast (1:1) (dashed orange), one myocyte under PeAF coupled to three fibroblasts (1:3) (dotted yellow), and one myocyte under PeAF coupled to nine fibroblasts (1:9) (dotted-dashed purple).

When fibroblasts were coupled to a single myocyte, we analyzed the effect of their electrophysiological characteristics (RMP_f_ and C_mf_) on myocyte electrical behavior (see Figure 2). When the number of coupled fibroblasts increased, the myocyte RMP became more depolarized and moved closer to the value of RMP_f_. A higher C_mf_ value seemed to further reduce myocyte APD when RMP_f_ was -45 mV.

**Figure 3:**
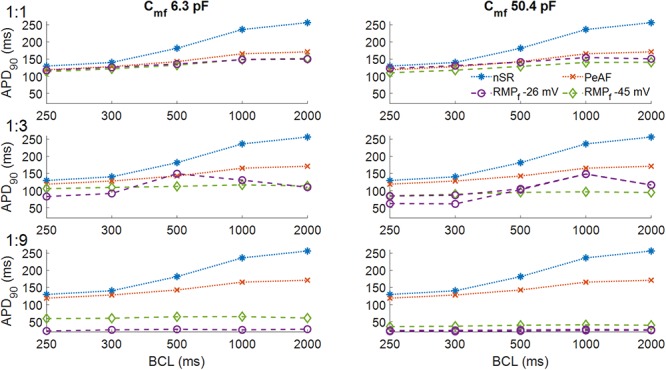
Action potential durations of myocytes in normal sinus rhythm (nSR), under persistent atrial fibrillation electrical remodeling (PeAF) and after being coupled to fibroblasts with a resting membrane potential (RMPf) of –26 or –45 mV in RA (baseline model). In the first column the membrane capacitance of fibroblasts is 6.3 pF and in the second column 50.4 pF. One myocyte was coupled to one fibroblast (1:1) (first row), three fibroblasts (1:3) (second row), and nine fibroblasts (1:9) (third row).

Figure 3 shows the effect of myocyte fibroblast coupling at different BCLs. As can be seen in Panel A, at a BCL of 300 ms myocyte AP was barely affected by coupling 1 fibroblast. APD_90_ was very slightly reduced at both RMP_f_ values (purple and green traces in panel A), for all BCLs.

In panel B at higher C_mf_ the effect on APD_90s_ was similar to Panel A, except for the fact that an RMP_f_ of -26 mV, APD_90_ was slightly higher than for an RMP_f_ of -45 mV, for all BCLs. As the number of fibroblasts increased (Panels C–F), the effects changed with the electrical characteristics of the fibroblasts and the BCL. Panels C and D show that for a different RMP_f_, APD_90_ changed with BCL. Longer APD_90s_ were obtained for BCLs of 500 ms and 1000 ms for a RMPf of -26 mV. In Panel D, for a BCL of 300 ms, APD alternans arose, and APD_90_ increased with higher BCL at an RMPf of -26 mV. When the number of coupled fibroblasts increased (Panels E and F), APD_90_ was reduced to 50 ms for -45 mV and all BCLs and to less than 40 ms for an RMP_f_ of -26 mV.

**Figure 4:**
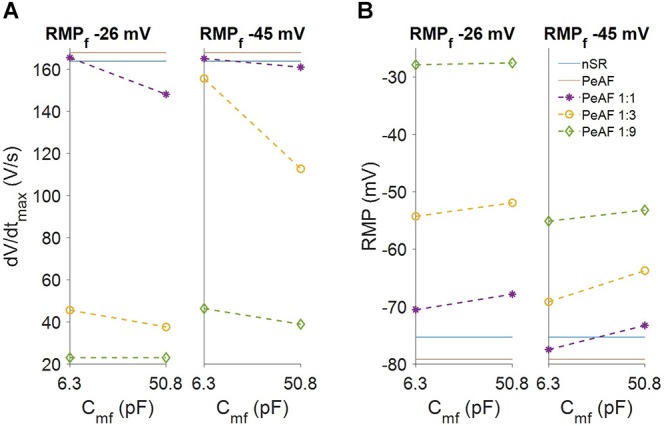
Fibroblasts coupling effect on myocyte electrophysiology. **(A)** Myocyte maximum upstroke velocity (dV/dt_max_). **(B)** Myocyte resting membrane potential (RMP). The levels for myocytes in normal sinus rhythm (nSR) and persistent atrial fibrillation (PeAF) conditions are given in blue and red, respectively. Discontinuous lines are myocytes in PeAF coupled to one fibroblast (1:1), 3 (1:3), or 9 (1:9).

We also analyzed myocyte RMP and dV/dt_max_ (see Figure 4). Myocyte RMP (Panel B) changed similarly when C_mf_ increased for both RMP_f_ values (-26 mV and -45 mV). Increasing the number of fibroblasts moved the myocyte RMP closer to RMP_f_. The most significant change was in the dV/dt_max_ (Panel A), which was much lower at an RMP_f_ of -26 mV than the reduction obtained at an RMPf of -45 mV. When fibroblasts couple to myocytes their RMP_f_ move closer to each other and more Na^+^ channels are available, although myocyte Na^+^ channel availability can be reduced due to a more depolarized RMP according to the number of coupled fibroblasts.

**Figure 5:**
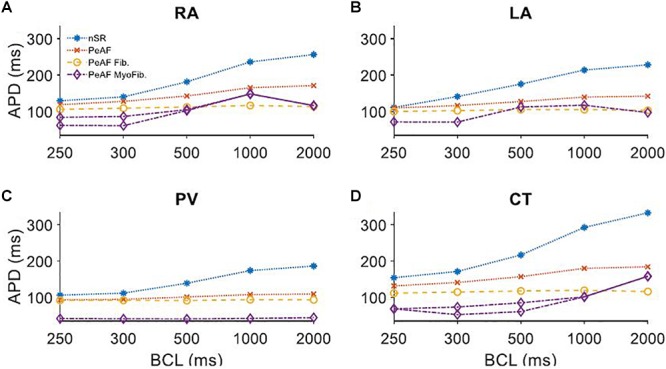
Myocyte action potential duration (APD) of four different regions of the atria under persistent atrial fibrillation electrical remodeling (PeAF) coupled to three fibroblasts (Fib) or myofibroblasts (MyoFib) stimulated at different basic cycle lengths (BCLs). **(A)** Right atria (RA), **(B)** Left atria (LA), **(C)** Pulmonary vein (PV), and **(D)** Crista terminalis (CT).

The experimental data agree with previous simulation studies in which fibroblasts presented an RMP of around -45 mV and a C_mf_ of 6.3 pF, while myofibroblasts have a more depolarized RMPf of -26 mV and a C_mf_ of 50.4 pF (Sridhar et al., 2017). In all the different atrial zones we analyzed the effects of coupling three fibroblasts or three myofibroblasts to a single myocyte (see Figure 5).

Panel A in Figure 5 shows how APD_90_ was reduced when fibroblasts were coupled for all BCLs. When myofibroblasts were coupled, for a BCL of 300 ms APD alternans arose and for a BCL of 1000 ms APD_90_ increased. Panel B shows APD_90s_ in LA for different BCLs and similar fibroblast and myofibroblasts effects. The reduced APD_90_ in the PV case (Panel C) seems to be more pronounced in myofibroblast coupling. CT alternans also arose when myofibroblasts were coupled.

### 1D Strand Simulations

**Figure 6:**
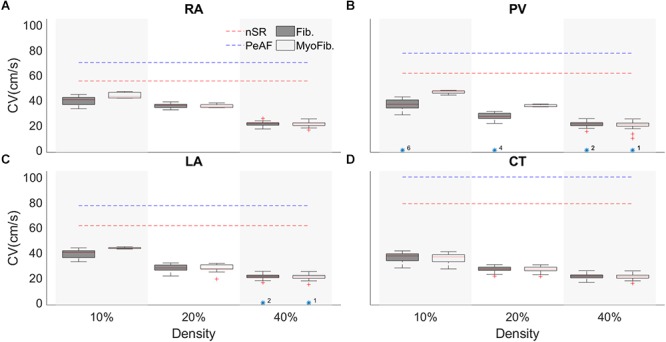
Conduction velocity (CV) of a tissue strand in four different regions of the atria with random fibroblasts (Fib) or myofibroblast (MyoFib) distribution and different fibroblast densities. For each density 100 random configurations were simulated. CV measurements are represented in boxplots. **(A)** Right atria (RA). **(B)** Pulmonary vein (PV), **(C)** Left atria (LA), and **(D)** Crista terminalis (CT).

Conduction velocity in the atria changes locally according to the region’s characteristics, as shown in Figure 6. During nSR and without fibroblasts, the RA has a CV of 70 cm/s, CT has a CV of 100 cm/s, LA has a CV of 70 cm/s, and PV has a CV of 80 cm/s (blue discontinuous lines) (Ferrer et al., 2015). AP propagation along the strand was affected by PeAF electrical remodeling (red discontinuous lines) and CVs dropped significantly. At higher fibroblast (yellow) or myofibroblasts (green) densities in the strand, CV dropped. Boxplot measurements of the CV are represented for the 100 random distributions of fibroblasts for each density (10, 20, and 40%). The region with the greatest differential effect on CV was the PV, depending on whether the distribution was with fibroblasts or myofibroblasts. PV also experienced conduction blocks in some of the random distributions (indicated by yellow asterisks). Conduction block was also seen in LA at a density of 40% in some distributions.

### 2D Tissue Simulations

**Figure 7:**
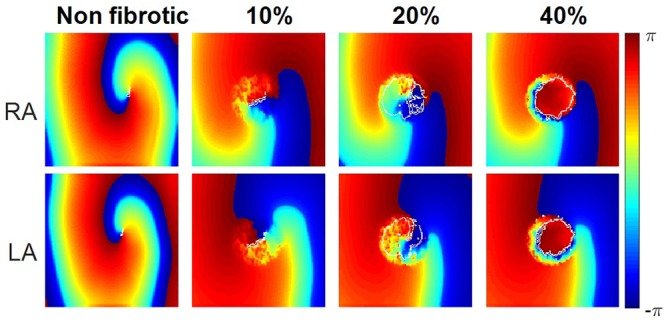
Instantaneous phase maps and phase singularities (in white) for different densities of myofibroblasts (non-fibrotic, 10, 20, and 40%) in the right atria (RA) and left atria (LA) under conditions of persistent AF remodeling.

The 2D atrial tissue electrical activity in PeAF with different myofibroblast densities was analyzed to assess vulnerability to reentry. Figure 7 shows snapshots of phase maps (taken at the same time). Re-entrant circuits can be seen in the RA (top panels) and in the LA (bottom panels) in PeAF remodeling and increasing levels of myofibroblast density from left to right (membrane potential snapshots can be seen in Supplementary Figure S4). In the absence of myofibroblasts a functional reentry was obtained in RA and LA. The tip of these rotors (Figure 7, first column), corresponding to the PS superimposed in white on the phase maps, describes a regular circular path, which agrees with the results obtained in previous simulation studies (Wilhelms et al., 2013). In LA, the rotor tip describes a smaller path due to the shorter wavelength caused by shorter LA ERP (Fernandez-Lozano et al., 2006; Krueger et al., 2012; Ferrer et al., 2015).

When myofibroblasts were present in the center of the tissue (see section “Materials and Methods”), the obstacle altered the reentrant activity. Small percentages of myofibroblasts (10–20%) allowed the wavefront to propagate through the fibrotic region, but the electrophysiological heterogeneities of myofibroblasts and myocytes caused wavebreaks, which were detected as PSs (quantified in Supplementary Table S3). However, propagation in the fibrotic region was practically blocked when myofibroblast density was raised to 40%, which produced an anatomical reentry surrounding the fibrotic obstacle. Since the wavefront did not propagate through the fibrotic region the number of wavebreaks was significantly reduced, as were the number of PSs detected (see Supplementary Table S3).

**Figure 8:**
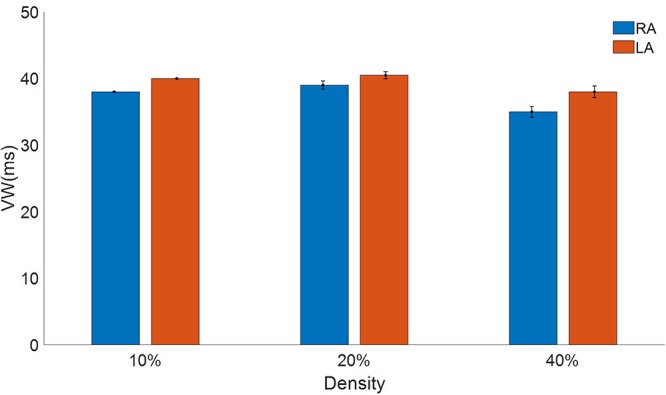
Vulnerable window to reentry in atrial tissue with different densities of myofibroblasts indicated in percentage for the right atrial (RA) in blue and the left atrial (LA) in red subject to persistent atrial fibrillation remodeling.

The myofibroblasts in the tissue increased reentry vulnerability, measured as the vulnerable window (VW), a time interval for which premature S2 stimulation generates a reentry (Figure 8). In the RA the vulnerable window in the absence of myofibroblasts was 37 ms. When myofibroblasts density was raised to 10%, the VW increased to 38 ± 0.0 ms. VW also increased (39 ± 0.63 ms) when density was raised to 20%, but at 40% VW dropped below the control value (35 ± 0.82 ms). Interestingly, LA was more sensitive to myofibroblasts with a larger VW than the RA. The LA VW in the absence of myofibroblasts was 40 ms. When myofibroblast density was raised to 10%, VW rose to 40 ± 0.0 ms, at 20% it increased to 40.5 ± 0.53 ms and at 40% it dropped to 38 ± 0.88 ms.

## Discussion

Computational modeling was used to investigate the effect of coupling fibroblasts and myofibroblasts to myocytes in four different regions of the atria during persistent AF. The study’s major findings can be summarized as follows: (i) myocyte-fibroblast coupling heterogeneously shortens myocyte APD and depolarizes the myocyte resting membrane potential in the 4 different atrial regions These effects strongly depend on the fibroblast electrophysiology. (ii) Fibroblasts, and specially myofibroblasts, introduce heterogeneities in the atrial substrate which alter the propagation of the AP, slowing conduction velocity. (iii) Fibroblasts-myofibroblasts change the atrial substrate during PeAF, which alters the vulnerability to re-entries and the vulnerable window presents a biphasic behavior related to myofibroblast density. These results suggest that the heterogeneity of the atrial tissue in the presence of fibroblasts/myofibroblats promotes reentrant events and alters the dynamics of arrhythmogenic propagation.

### Heterogeneous Effects of PeAF Remodeling and Electrical Coupling of Fibroblasts in Atrial Tissues

Atrial substrate is differently affected by PeAF remodeling and by the presence of fibroblasts, due to the electrophysiological heterogeneity of the different atrial regions. Our results from isolated single cells show differences in the RMP, dV/dt_max_, and APD for the four different atrial regions (RA, LA, CT, and PV) in nSR and in PeAF. These differences are in agreement with the simulations carried out by Krueger et al. (2013), who reported the differential effects of AF remodeling in the different atrial regions. It has to be noted that in contrast to the Krueger study, our model presents a long-term stability in all regions in single-cell and tissue simulations and also considers the effect of fibroblast coupling. To our knowledge, this is the first simulation study including the three components (atrial heterogeneity, AF remodeling, and fibroblasts) using a detailed electrophysiological AP model for fibroblasts and focusing on the analysis of the different effects exerted by fibrosis in the different atrial regions. A recent study by Roney et al. (2018) showed that high PS density in the PVs favored the effectiveness of PV isolation in ablation procedure. Their model also considered electrophysiological remodeling in AF, electrophysiological heterogeneities in different atrial regions, and fibrosis was simulated by changes in tissue conductivity. In a previous study (Roney et al., 2016), the same group modeled fibrosis by different methods and did not consider either electrophysiological heterogeneities in the atrial regions or AF remodeling to determine how different fibrosis models could affect rotor dynamics.

Different experimental studies show that atrial fibroblasts have a different electrophysiology from ventricular fibroblasts (MacCannell et al., 2007; Burstein and Nattel, 2008; Poulet et al., 2016; Salvarani et al., 2017). Morgan et al. (2016) found that fibroblast electrophysiology changes the dynamics of an arrhythmic process and provides relevant information on the effect of myocyte-fibroblast coupling in the atria. Our results indicate that RMPf, C_mf_, and the number of coupled fibroblasts altered the behavior of myocytes AP, as was found in previous simulation studies (Maleckar et al., 2009; Koivumäki et al., 2014a; Sridhar et al., 2017). Furthermore, in the present study we found that introducing I_Na_ current into the fibroblast model had an interesting effect; due to the high RMP of isolated fibroblasts I_Na_ current was blocked but when fibroblasts were coupled with myocytes I_Na_ channels became available. Additionally, myocyte-fibroblast coupling led to a partial inactivation of the myocyte I_Na_ due to the higher RMP in the myocyte.

Fibroblast electrophysiology (RMPf and C_mf_) changes myocyte AP characteristics (Jacquemet and Henriquez, 2008, 2009; Maleckar et al., 2009). Our simulation results also show that electrical coupling with myocytes increases atrial electrophysiological heterogeneity. Changes in the BCL altered the behavior of the coupled cells, with different responses in different regions. Interestingly, myofibroblast -myocyte coupling in regions with higher I_K1_ and I_CaL_ (RA and CT) exhibited more sensitivity to changes in frequency, while regions with smaller I_K1_ and I_CaL_ (PV) developed no AP for any of the BCLs. In contrast to McDowell et al. (2013), we defined different electric characteristics for the atrial myofibroblasts, which have a different effect on myocyte AP. Myofibroblasts act as the current source, raising the myocyte RMP (Jacquemet and Henriquez, 2007) according to the number of coupled myofibroblasts (Maleckar et al., 2009; Koivumäki et al., 2014a), thus leading to a partial inactivation of the myocyte I_Na_ current (see Supplementary Figure S5).

### Heterogenous Effects of Fibrosis During PeAF in Atrial Tissues

Structural remodeling of the cardiac tissue contributes to reducing conduction velocity, delaying regional functional activations, and increasing structural heterogeneities, which are important factors for establishing a re-entrant driver or conduction block (Camelliti et al., 2005). Our results show that fibroblasts and myofibroblasts can alter the activation time in a 1D tissue strand, in agreement with different studies (Sachse et al., 2008; Xie et al., 2009). We implemented one hundred random configurations for different fibroblast/myofibroblast densities in the four atrial regions. Zhan et al. (2014) showed that fibroblasts can alter the CV and can lead to blocks in conduction with fibrosis densities of 40 and 45%, our results showed that a high density (40%) led to conduction blocks in the LA and that the PV was the most sensitive region to the presence of fibroblasts-myofibroblasts. Similarly, in an experimental study Miragoli et al. (2006) showed that myofibroblast proliferation changed the tissue conduction velocity and myocyte dV/dt_max_. Our results showed a reduction in CV, in agreement with several other experimental and simulation studies that found that fibroblasts-myofibroblasts can establish an electric coupling with myocytes, reducing their dV/dt_max_ and activation time, reflected in reduced CV (Miragoli et al., 2006; Rohr, 2009; Xie et al., 2009; Yue et al., 2011). We also found a monotonic reduction in all four atrial regions.

Tanaka et al. (2007) have shown that local fibrosis distribution reduces CV in the different atrial regions, in agreement with our results, which showed a reduced CV with a tendency to homogenize in all four atrial regions. CV heterogeneity is responsible for giving the atria the characteristic activation times (Nguyen et al., 2012); if all the regions were to have a homogenous CV, this might induce the appropriate conditions for reentrant rhythms and conductions blocks (Gaspo et al., 1997). As the fast conduction systems’ (CT) conduction velocity was significantly reduced, this may be an interesting mechanism for AF in the right atrium.

### Vulnerability to Reentry During PeAF in Fibrotic Tissue

Structural remodeling and endo-epicardial dissociation alter the atrial substrate and could produce macroreentries and focal activity (Everett and Olgin, 2007; Verheule et al., 2014). When, we analyzed the propagation in different regions of the atria and at different myofibroblast densities, we found that a low myofibroblasts percentage increased the number of PSs due to the wavebreaks. However, at higher percentages, propagation through the fibrotic zone was blocked, the number of wavebreaks and PSs decreased, and an anatomical reentry was anchored around the fibrotic zone, in agreement with previous studies (McDowell et al., 2013; Roney et al., 2016). Several simulation studies have also shown that reentry dynamics is altered by heterogeneities of the AP in the cardiac tissue (Colman et al., 2014; Varela et al., 2016), the presence of fibroblasts (Ashihara et al., 2012; Gomez et al., 2014a; Morgan et al., 2016), and that PSs increase in the zones with fibroblasts (Saha et al., 2018).

Waks and Josephson (2014) demonstrated that the rotation dynamics depends on the atrial tissue (RA or LA) and its electrophysiological characteristics, as did we in the present study, in which vulnerability to reentries and the dynamics of the rotation depended on the atrial region. LA presented slightly higher VWs, due to its shorter APD.

Gomez et al. (2014b) showed that the density of fibroblasts had a biphasic impact on the ventricular vulnerable window for reentry, while our results showed the same VW biphasic behavior for the first time in atrial tissue. Krul et al. (2015) found that local fibrosis is associated with reentrant activity, comparable to our results at low fibroblast density (10%), which can be considered as a region of local diffuse fibrosis, presented higher tissue vulnerability and resulted in multiple wavebreaks. When myofibroblast density was raised (20%), tissue vulnerability to reentry rose and conduction blocks occurred. However, at higher densities (40%) the conduction blocks also occurred but VW dropped, as was found by Campos et al. (2018). This suggests that myocyte-fibroblast coupling in PeAF plays an important role in AF (Morgan et al., 2014), with different effects in different atrial regions.

### Limitations

Several limitations must be considered when drawing conclusions from the present study. Firstly, we did not consider the effect of electrical remodeling due to cytokines like TGF-B, which have been reported during structural remodeling and are known to affect myocyte AP (Burstein and Nattel, 2008; Nattel et al., 2008; Zahid et al., 2016).

Secondly, as fibroblast electrophysiology and differentiation into myofibroblasts still remain unclear, the models we used should be considered as an approximation (Nattel, 2018). Myofibroblast electrophysiological characteristics are still not well understood. Furthermore, it is important to highlight that although animal models and *in vitro* experiments prove the existence of electrotonic coupling between myocytes and fibroblasts (Kohl et al., 2005; Miragoli et al., 2006; Grand et al., 2014; Salvarani et al., 2017), this has not been reported in humans *in vivo*. However, human cardiac tissue with fibrosis presents altered electrical behavior (Krul et al., 2015; Fukumoto et al., 2016).

Thirdly, the pulmonary vein AP model was built on the basis of the reported experimental data for dogs and sheep APD_90_ and RMP. Electrophysiological data from human isolated myocytes from PV are still unavailable (Ehrlich et al., 2003).

Fourthly, while AF mechanisms are still unclear, there are different pathways by which reentrant drivers can be generated and maintained. Several studies have shown how non-myocyte cells like fibroblasts, macrophages (Hulsmans et al., 2017) and adipocytes (De Coster et al., 2018) alter the atrial substrate and promote arrhythmias. The effects of non-myocytes or endo-epicardial dissociation have not been considered in the present study, although these are factors which might alter the reentry dynamics and would be interesting to analyze in future studies (Verheule et al., 2014).

Fifthly, we are aware that fibrosis is a complex structure involving different actors such as collagen deposition, inflammatory cytokines and proteins which may alter the myocyte electrophysiology. There are different approaches to simulating the fibrotic regions such as non-conductive elements (Ten Tusscher et al., 2004), the paracrine effect changing the myocyte ion channel conductance (Zahid et al., 2016) and coupling to elements with a static RMP (Majumder et al., 2011) and using ionic models to describe the electrophysiology of fibroblasts (McDowell et al., 2013; Morgan et al., 2016; Saha et al., 2018).

Finally, we did not include the effect of different sizes or locations of fibrotic regions in the tissue. The fibrotic regions can in fact attract rotors (Roy et al., 2018), and future studies in this line would shed light into the mechanisms of chaotic rhythms when fibroblasts proliferate. Additionally, 3D simulations have shown how fibroblasts and non-conductive areas can modify the dynamics of the reentry (McDowell et al., 2013; Morgan et al., 2014). Zahid et al. (2016) introduced the paracrine effect, modifying myocyte electrophysiology in the fibrotic region in 3D simulations reducing the AP duration and the conductivity of the fibrotic region. We are also aware that 3D simulations include further structural details. The work by Ferrer et al. (2015), among others, presents a highly detailed atrial model. However, these simulations have a high computational cost while 2D simulations are computationally more efficient and provide a detailed insight into arrhythmia dynamics. Despite these limitations, we consider that our simulations represent a realistic heterogeneous PeAF remodeling (electrophysiological and structural) scenario in the different atrial regions and contribute a great deal to the understanding of AF mechanisms.

## Conclusion

The results of the present simulation study show that fibroblast electrophysiology alters myocyte AP characteristics and leads to slower conduction velocity in atrial tissues affected by PeAF. These changes are heterogeneous within the different atrial regions.

Myocyte-fibroblast coupling creates a substrate in which the dynamics of arrhythmic reentries changes with the fibroblast density. By increasing the density of fibroblasts, reentries evolve from functional to anatomical around the obstacle formed by the fibrotic region. We also observed biphasic behavior of tissue vulnerability to reentries. Low myofibroblast densities (10 and 20%) increase the vulnerability to reentry, while a higher density (40%) reduces tissue vulnerability.

## Author Contributions

JoS, JaS, and BT contributed to the conception and design of the study. JG, LR, and LM-M contributed to the development of the model. JoS performed the computational simulations and JoS, JaS, and BT performed the analysis and interpretation of the results. All authors contributed to drafting of the manuscript and all revised, and approved the submitted version.

## Conflict of Interest Statement

The authors declare that the research was conducted in the absence of any commercial or financial relationships that could be construed as a potential conflict of interest.
