# Supplementary material for: Heterogeneous Effects of Fibroblast-Myocyte Coupling in Different Regions of the Human Atria Under Conditions of Atrial Fibrillation
Source: Front Physiol. 2019 Jul 4;10:847. doi: 10.3389/fphys.2019.00847 (PMC6620707; doi:10.3389/fphys.2019.00847)
Supplement: Supplementary file 1 [file Table_1.DOCX]

**SUPPLEMENTAL MATERIAL**

**Supplemental Methods:**

The equations below correspond to the sodium current in the fibroblast membrane (I_Na-fib_) formulated by Koivumaki et al. (2014) and updated to fit experimental data from Poulet et al. (2016) and Salvarani et al. (2017) data. The modified parameters can be found in table S1.

T = 306.15 K

F = 96487.0 C/mol

R = 8314.0 mJ/(mol*K)

q_10tauNa_ = 3

$$I_{Na-fib}= P_{Na-fib}*m^{3}*\left( 0.9*h_{1}+0.1*h_{2} \right)*{[Na}_{o}]*V_{fib}*\frac{F^{2}}{R*T}*\frac{e^{\left( \frac{{(V}_{fib}-E_{Na-fib})*F}{R*T} \right)}-1}{e^{\left( \frac{V_{fib}*F}{R*T} \right)}-1}$$

$$m_{inf}=\frac{1}{1+e^{\left( \frac{V_{fib}+m_{1/2}-0.465*(T-294.15)}{m_{k}} \right)}}$$

$$h_{inf}= \frac{1}{1+e^{\left( \frac{V_{fib}+h_{1/2}-0.785*(T-294.15)}{h_{k}} \right)}}$$

$${tau}_{m}= \frac{1}{q_{10tauNa}}*0.000042*e^{-\left( \frac{V_{fib}+25.57}{28.8} \right)^{2}}+0.000024$$

$${tau}_{h1}= \frac{1}{q_{10tauNa}}*\frac{0.03}{1+e^{\left( \frac{V_{fib}+35.1}{3.2} \right)}+0.0003}$$

$${tau}_{h2}=\frac{1}{q_{10tauNa}}*\frac{0.12}{1+e^{\left( \frac{V_{fib}+35.1}{3.2} \right)}+0.003}$$

**Table S1.** Parameter modification for fibroblast sodium current.

| P_Na-fib_ | 0.0000225 |
| --- | --- |
| m gate activation (m_1/2_) | 29.9 |
| m gate steepness coefficient (m_k_) | -3.1 |
| h gate activation (h_1/2_) | 64.7 |
| h gate steepness coefficient (h_k_) | 6.9 |

**Table S2.** Parameter modification in Koivumaki et al. (2014) fibroblast formulation to achieve a fibroblast resting membrane potential of -26 mV.

| g_Kv_ | 1.75 |
| --- | --- |
| r_kv_ | 15 |
| s_kv_ | 18 |
| g_K1_ | 3 |
| g_NaK_ | 2.75 |
| g_Nab_ | 8.1275 |

| 1. **0D** | 1. **1D** | 1. **2D** |
| --- | --- | --- |
| 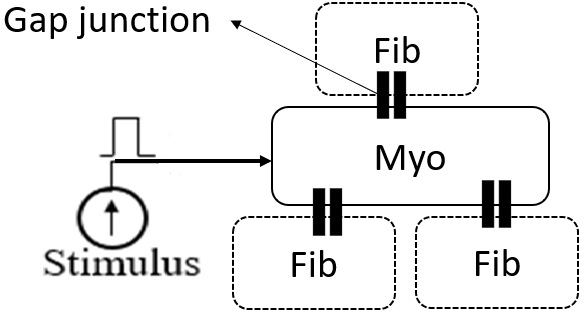 | 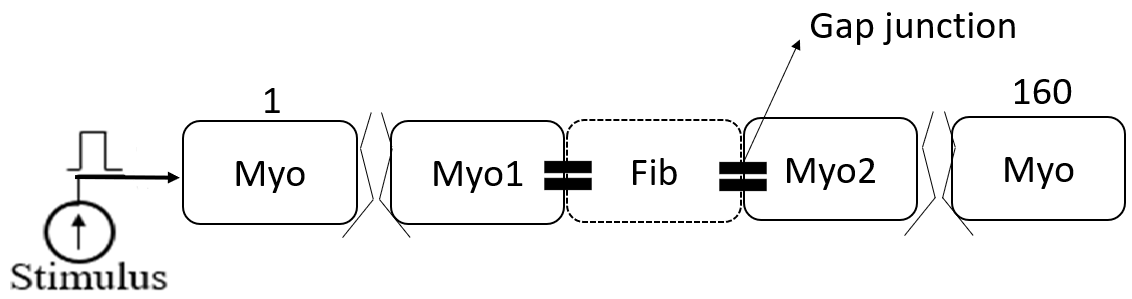 | 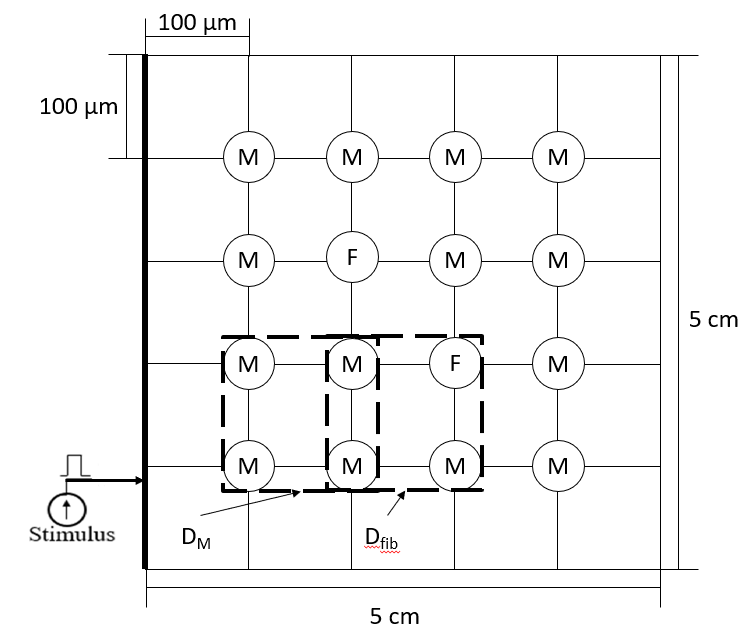 |

**Figure S1.** 0D, 1D, and 2D configurations of myocyte-fibroblast coupling. A) Configuration of a single myocyte coupled with 1 – 9 fibroblasts. B) 1D strand configuration using a uniformly distributed random density of fibroblasts (10%, 20%, 40%). The stimulation was applied to the first element with an amplitude of twice the threshold. C) 2D tissue with uniformly distributed random fibroblasts density (10%, 20%, 40%) in a defined region. The initial stimulation was applied to the left edge of the tissue.


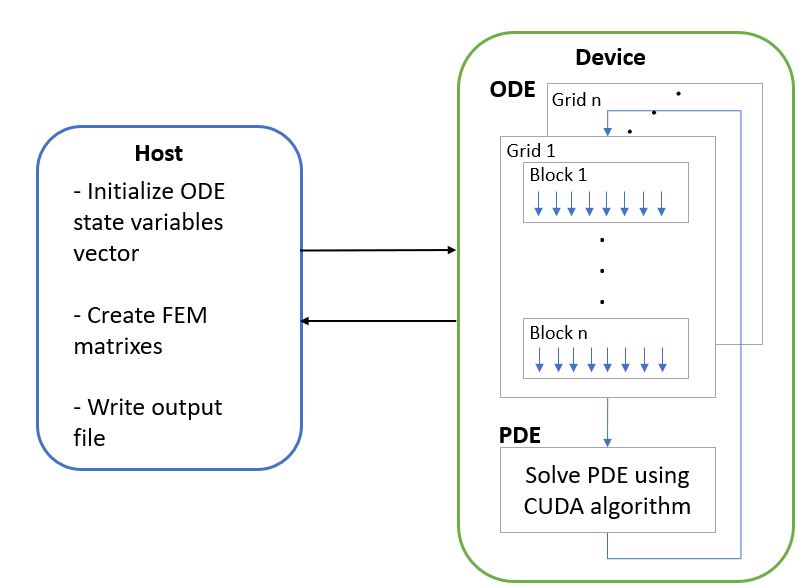


**Figure S2.** Diagram of the algorithm used to execute 1D simulations. ODE state variables vector was initialized in host (CPU) and copied to the device (GPU). In the device a grid was created containing the number of blocks equivalent to the number of nodes of the mesh. Each block has several threads equal to the number of state variables of the ionic model. FEM matrixes were assembled using Eigen library and were transferred to the device. Then PDEs were solved using linear solvers implemented in the CUDA library. The solution vector of the PDEs was transferred to host were the output file was written.

**A) 1D**

**10%**
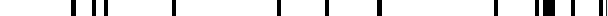


**20%**
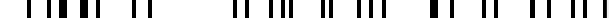


**40%**
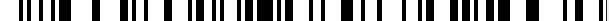


**B) 2D**

**10% 20% 40%**


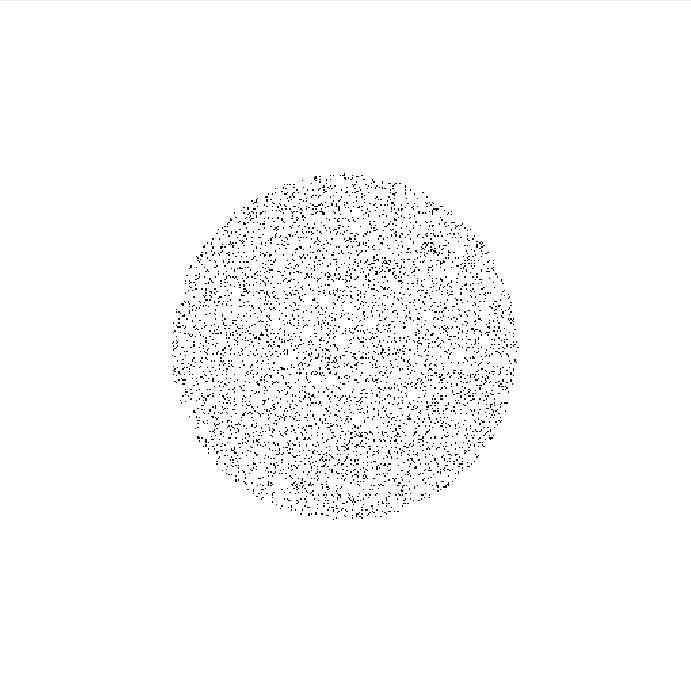

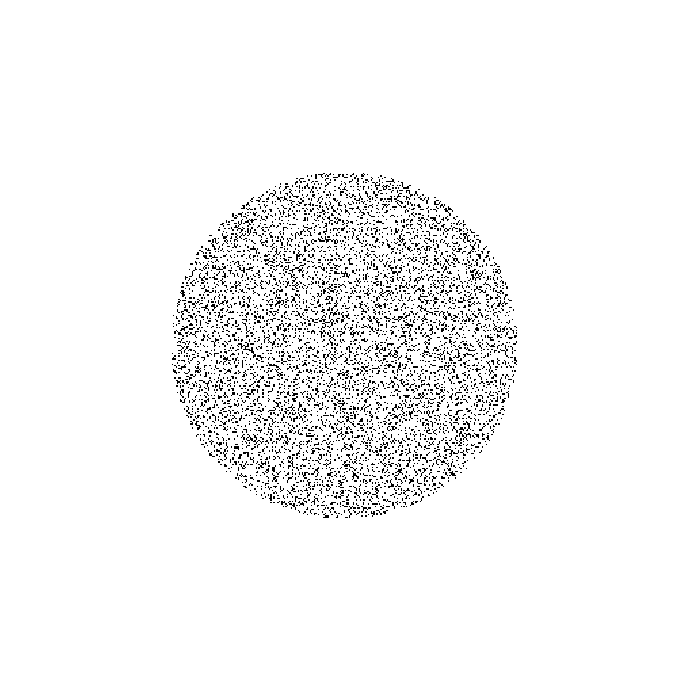

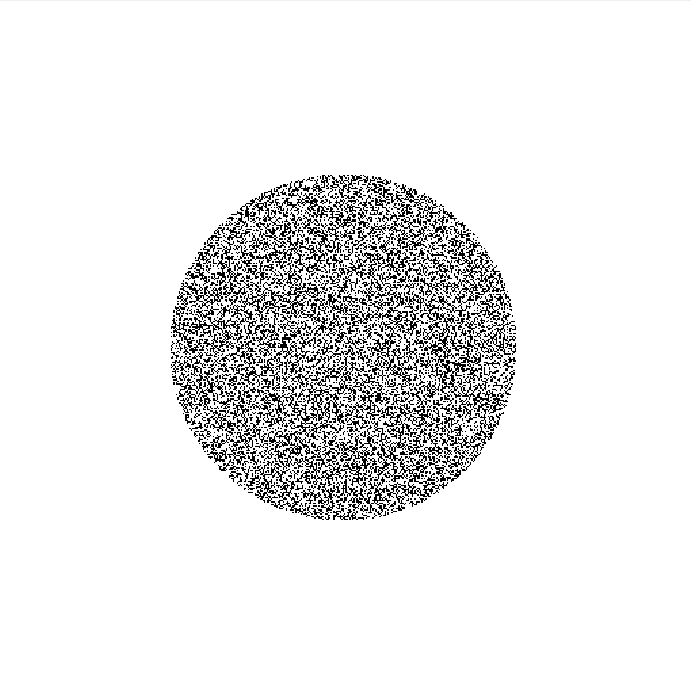


**Figure S3.** 1D and 2D uniformly distributed random fibroblasts densities (10%, 20%, and 40%). **A)** One of one hundred 1D strand uniformly distributed random fibroblast for each of the densities considered. **B)** Uniformly distributed random fibroblasts in a 2cm diameter circular region for the different densities.


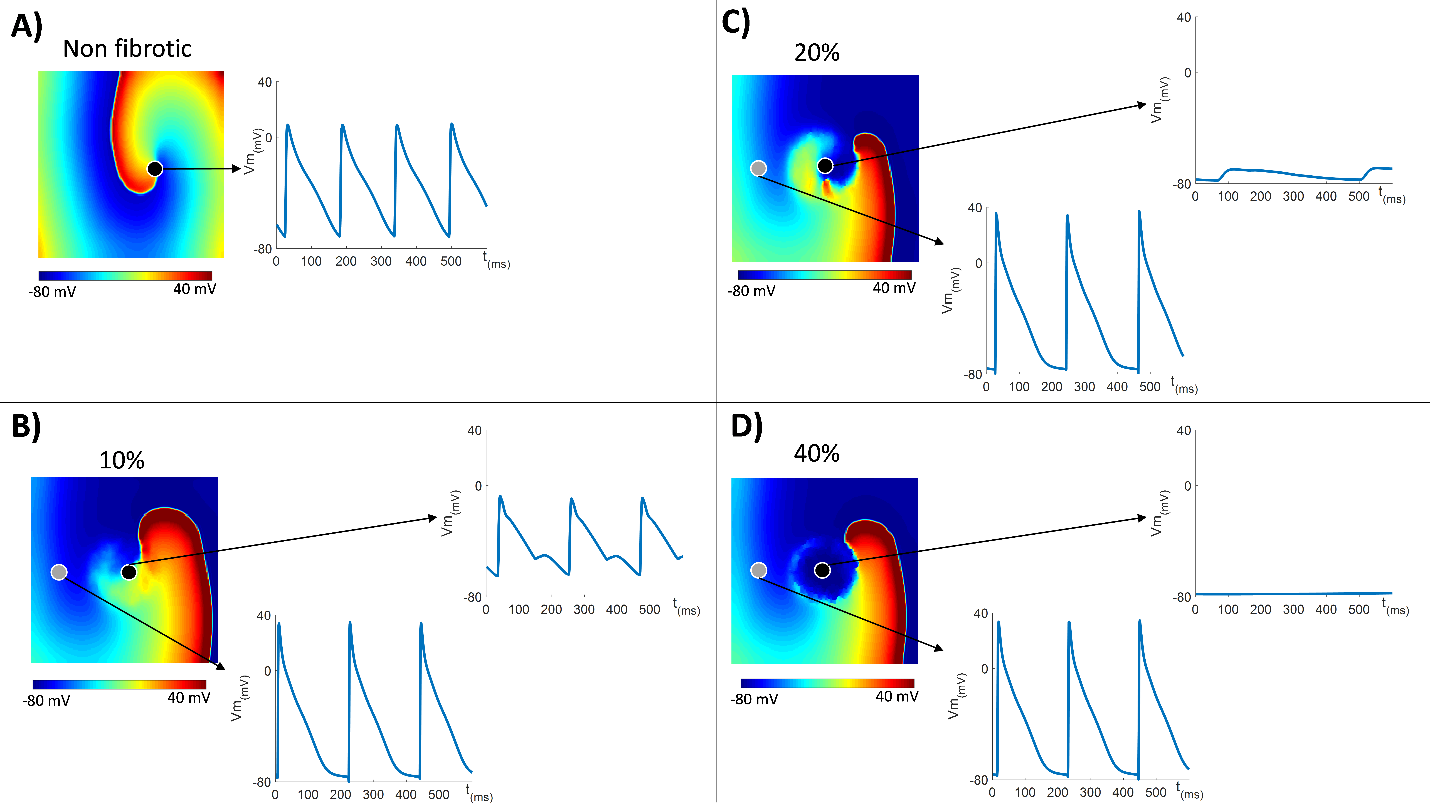


**Figure S4.** Membrane potential maps and action potentials in the center (black dot) of the tissue and in the left part of the tissue (gray dot) out of the fibroblast’s distribution region. **A)** Top left panel, non-fibrotic tissue. **B)** Bottom left panel tissue with 10% of fibroblast density. **C)** Top right panel tissue with 20% density of fibroblasts, at the center of the tissue we observed no action potential. **D)** Bottom right panel tissue with 40% of density, in the center of the tissue there is a slightly variation of the RMP while outside we have a full action potential.

**Table S3.** Quantification of PSs in different regions of the atria in control, with 10%, 20%, and 40% of myofibroblasts uniformly distributed. PSs were computed during 4 seconds of simulation.

|  | **Number of PS** | |
| --- | --- | --- |
|  | **RA** | **LA** |
| control | 3234 | 3600 |
| 10% | 4271 | 6112 |
| 20% | 14312 | 11954 |
| 40% | 10660 | 11241 |

**Figure S5.** Effect fibroblast-myocyte coupling in persistent atrial fibrillation (PeAF). First and second row correspond to values of fibroblasts resting membrane potential (RMPf) of -26 mV and -45 mV, respectively, for a fibroblast membrane capacitance (Cmf) of 6.3 pF (first column) and 50.4 pF (second column). The different traces correspond to the availability of the sodium channel of an isolated myocytes in PeAF (blue), one myocyte in PeAF coupled to 1 fibroblast (1:1) (dashed orange), one myocyte in PeAF coupled to 3 fibroblasts (1:3) (dotted yellow), and one myocyte in PeAF coupled to 9 fibroblasts (1:9) (dotted-dashed purple).

**References:**

Koivumäki, J., Clark, R. B., Belke, D., Kondo, C., Fedak, P., Maleckar, M. M., et al. (2014). Na+ current expression in human atrial myofibroblasts: Identity and functional roles. *Front. Physiol.* 5 JUL, 1–14. doi:10.3389/fphys.2014.00275.

Poulet, C., Künzel, S., Büttner, E., Lindner, D., Westermann, D., and Ravens, U. (2016). Altered physiological functions and ion currents in atrial fibroblasts from patients with chronic atrial fibrillation. *Physiol. Rep.* 4, e12681. doi:10.14814/phy2.12681.

Salvarani, N., Maguy, A., De Simone, S. A., Miragoli, M., Jousset, F., and Rohr, S. (2017). TGF-β1(Transforming Growth Factor-β1) Plays a Pivotal Role in Cardiac Myofibroblast Arrhythmogenicity. *Circ. Arrhythmia Electrophysiol.* 10. doi:10.1161/CIRCEP.116.004567.
